# Science quality and the value of inventions

**DOI:** 10.1126/sciadv.aay7323

**Published:** 2019-12-11

**Authors:** Felix Poege, Dietmar Harhoff, Fabian Gaessler, Stefano Baruffaldi

**Affiliations:** 1 Max Planck Institute for Innovation and Competition, Munich, Germany.; 2 Institute for the Study of Labor (IZA), Bonn, Germany.; 3 Ludwig Maximilian University (LMU), Munich, Germany.; 4 Centre for Economic Policy Research (CEPR), London, UK.; 5 Technical University of Munich, Munich, Germany.; 6 University of Bath, Bath, UK.

## Abstract

Despite decades of research, the relationship between the quality of science and the value of inventions has remained unclear. We present the result of a large-scale matching exercise between 4.8 million patent families and 43 million publication records. We find a strong positive relationship between the quality of the scientific contributions referenced in patents and the value of the respective inventions. We rank patents by the quality of the science to which they are linked. Strikingly, high-ranking patents are twice as valuable as low-ranking patents, which, in turn, are about as valuable as patents without a direct science link. We show this core result for various science quality and patent value measures. The effect of science quality on patent value remains relevant even when science is linked indirectly through other patents. Our findings imply that what is considered excellent within the science sector also leads to outstanding outcomes in the technological and commercial realms.

## Teaser

Common quality measures for scientific work predict the commercial value of patents directly or indirectly derived from them.

## INTRODUCTION

The relationship between science and technology has been subject to intense discussions for centuries. Science was largely funded via patronage during the Renaissance, and separation of public funding for fundamental research and private industrial funding for applied research and commercial innovation efforts only emerged in the 19th century (*1*, *2*). Since the aftermath of World War II, policymakers have relied on the notion that science helps to generate knowledge and information that ultimately contributes to the emergence of new technical and organizational capabilities, improvements in quality of life, and economic growth (*3*). Vannevar Bush’s vision of a publicly funded science system that feeds into privately organized innovation channels became the blueprint for most of the Western national systems of science funding, research and development, and innovation. This notion has recently come under scrutiny again, as voters have increasingly been demanding evidence on the benefits of science spending. For policymakers and scientists alike, it is tantamount to improve the understanding of the impact of science on technical progress and innovation.

The most pertinent form of output delivered by the science sector is publications, which are known to vary widely in quality. While some scientific publications will reach and inspire large numbers of researchers, others are never read or referenced. Measures of scientific quality, such as citation counts or impact factors, are used to make this heterogeneity visible and have become increasingly important in the governance of the science sector. Science governance and science funding seek to promote excellent over more mediocre science output by allocating resources to those researchers and institutions from which outstanding results can be expected.

However, it has been argued that this logic does not take tangible results from technology transfer and commercialization into account. Science is inward-looking, according to these voices. This raises the question as to what extent science output that is considered “excellent” within the science sector can lead to outstanding outcomes in the technological and commercial realms. This paper seeks to contribute new insights into the understanding of this nexus.

We provide evidence that the quality of scientific publications—as commonly assessed in science via citations—is a strong predictor of their relevance for and impact on technology development as documented in patents. We document two main results. First, publications with high scientific quality are vastly more likely to be cited in patent documents and at a higher rate. This confirms the baseline results of previous research going back to Hicks *et al.* (*4*) on a substantially larger and more diverse dataset. Second, the value of patents that directly build on science increases monotonically with science quality. These results hold across scientific disciplines, technological areas, and time. Ahmadpoor and Jones (*5*) recently established that patents more closely related to science are more valuable. We confirm that closeness to science matters; however, this relationship is largely driven by the actual science quality. Considering both dimensions together provides the most comprehensive view of the science quality–patent value relationship.

### Data

Our analysis starts from the universe of scientific publications in Web of Science (WoS) from the year 1980 onward, corresponding to approximately 43 million scientific publications. In terms of patents, we consider a sample of more than 4.8 million patent families, comprising all patent families from the database DOCDB with at least one grant publication at the European Patent Office (EPO) or the U.S. Patent and Trademark Office (USPTO), with first filing date between 1985 and 2012 included. Subsequently, our unit of analysis is the patent family, to which we also interchangeably refer as “patents.” The patents protect inventions in developed countries with more than 1 billion inhabitants in total.

Patents reference various types of documents that relate to the protected invention by either determining novelty (prior art) or explaining the content of the underlying invention. These documents listed on the patent’s front page or in so-called search reports include not only other patents foremost but also frequently nonpatent literature (NPL) (*6*). A subset of the latter are references to scientific articles, which we dub scientific NPL (SNPL).

To link patents to publications, we leverage a highly precise and comprehensive match of NPL references in patents with scientific publications in WoS. The NPL references in patents that were successfully linked to scientific publications comprise our set of SNPL references. Around 0.9 million patents were linked to at least one scientific publication via a total of about 7.0 million SNPL references. Of all scientific publications, about 2.2 million figure in this list of SNPL references.

**Fig. 1:**
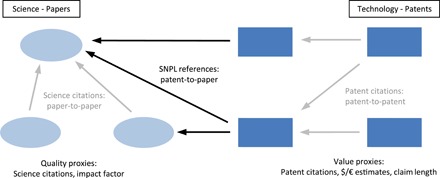
Setting: Domains of science (left), technology (right), and patent-paper references.

In our core set of analyses, we rely on established measures of scientific quality and patent value. The quality of scientific publications is measured by the number of citations from other scientific publications over a period of 3 years since publication. We define a patent’s SNPL science quality as the quality of the patent’s SNPL references. A patent can reference zero, one, or several scientific articles in the same way that a scientific article can be referenced by zero, one, or many patents. Figure 1 illustrates this setup. When more than one SNPL reference is present, we consider by default only the publication of the highest quality. Patent value is measured by the number of forward patent citations over a period of 5 years from the patent’s first filing date. We use citations by U.S. patents as our first measure of patent value. Our results are robust to alternative choices. We replace citations as science quality measure with the journal impact factor. We replace our aggregation method of the quality of multiple SNPL references with several other options. We replace U.S. patent citations as value measures with a host of alternatives. The Supplementary Materials provide further detailed information on data sources, discuss the use of citations as indicators of relatedness between technology and science, and elaborate on alternative measures of patent value and scientific quality that we use for robustness analyses.

## RESULTS

**Fig. 2:**
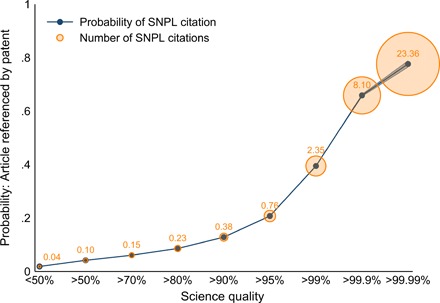
SNPL references by science quality. Science quality is the 3-year citation count from other scientific publications. The patent count is not conditional on appearing as an SNPL reference. Blue shaded areas show 95% confidence intervals around the mean. *N* = 42,962,463.

We first explore the selection of scientific publications into the patent realm, i.e., the relationship between science quality and the likelihood that a scientific publication is referenced in a patent. We look at the probability and intensity of referencing, i.e., if any and how many patented inventions refer to a given scientific contribution. We present results for publications below the median (all receiving zero science citations), for publications between the median and the 70th percentile, and the 80, 90, 95, 99 (top 1%), 99.9 (top 1 permille), and 99.99 (top 1 permyriad) percentiles of scientific quality. Figure 2 presents these results; the line plots the share of scientific publications appearing as SNPL references in at least one patent, and the size of the circles indicates the average number of times they appear as SNPL references.

We find a remarkably strong positive selection of scientific publications of high scientific quality into SNPL references. Below the median, scientific publications are almost never SNPL references. This number increases up to 40% at the top 1% of publications by scientific quality. A staggering majority of publications at the top 1 permille (>60%) and beyond the top 1 permyriad (80%) are referenced in the patents. The average number of times they appear as SNPL references in distinct patent families is 8.1 and 23.36, respectively. We emphasize that these results are not due to feedback from important patents to citations of the underlying science. By restricting our measure for scientific citations to the first 3 years after publication, we effectively exclude this bias.

In our main analysis, we investigate the extent to which SNPL science quality is a predictor of patent value. The main figures account for level differences across technology fields and over time: We estimate econometric models that absorb variation across these dimensions with pair-level fixed-effect (FE) controls and graphically present the resulting residual values. In effect, we transform deviations from the technology field and year-specific mean to deviations from the overall mean. In this way, the main results we present graphically account for structural changes over time across technological areas and constitute a baseline correlation with an immediate interpretation.

**Fig. 3:**
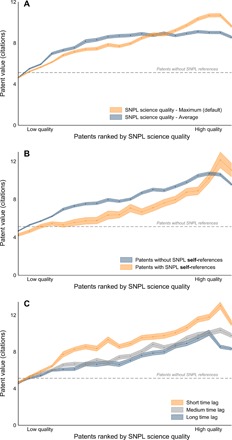
Patent value by SNPL science quality. SNPL science quality is the maximum 3-year citation count across scientific publications appearing as SNPL references in a patent. Patent value is measured as the 5-year count of patent forward citations by U.S. patents. Patent value and science quality are residualized using technology field × first filing year FEs. Shaded areas show 95% confidence intervals around the respective means. (**A**) When there are multiple patent-paper references, we, by default, use the highest-quality reference (orange). In comparison, we use the average quality (blue). (**B**) SNPL self-references of the highest-quality SNPL references are considered. (**C**) Time distance is measured as the lag between the first filing year of the patent and the publication year of the scientific publication in SNPL references with the highest science quality. *N* = 4,767,844 patents (948,006 with SNPL references).

The relationship between SNPL science quality and patent value is depicted in Fig. 3A. We plot the average patent value across the distribution of SNPL science quality. As a first measure of patent value, we use the number of patent citations from U.S. patents. Later on, we consider alternatives. As a benchmark level, the figure shows the average value of patents without any SNPL reference (dashed line). We contrast two possible aggregation methods of SNPL science quality. When a patent references multiple scientific articles, we use, in a first variant, the highest-quality reference (orange). Here, we juxtapose a second variant where we consider the average quality of all references. Top science matters much more, considering that scientific material beyond the highest-quality reference dilutes the science quality–technology value relationship. In the Supplementary Materials, we show that this extends to other aggregation methods that focus on the top of the quality distribution. Consequently, we continue by only considering the highest-quality SNPL reference.

Previous studies have shown that SNPL references or references to other technical literature are associated with higher-value patents (*5*, *7*, *8*). We are able to confirm this finding in our data: The value of patents with SNPL references is higher than or equal to that of patents without SNPL references for any level of SNPL science quality, except the very bottom.

Notably, SNPL science quality fully explains the difference in average value between patents with and without SNPL references. Patent value increases rapidly, and almost monotonically, for a higher level of SNPL science quality. Patents with SNPL references at the bottom of the SNPL science quality distribution are, on average, as valuable as patents without SNPL references. Compared to this group, patents at the top of the SNPL science quality distribution receive more than twice as many forward patent citations. This core result suggests that scientific activities of high quality may lead to the development of highly valuable technologies.

Sometimes, high-quality research and technology development are undertaken by the same individuals or organizations, which may drive the result. Inventors and scientists can perform scientific activities that may lead directly to both scientific and technological outcomes (*9*). Therefore, we complement this finding by exploring how our results vary when considering separately SNPL self-references, whether at the author or institutional level. Figure 3B describes the corresponding results. The line in orange indicates the patent value of patents with SNPL self-references, i.e., those that overlap at the individual or institutional level. The line in blue describes the value of patents excluding SNPL self-references. The latter presents close to identical results to those obtained in Fig. 3A. Note that for part of the SNPL science quality distribution, with the exception of the very top, patent value is higher when patents with SNPL self-references are excluded. The share of SNPL self-references is roughly similar and, if anything, tends to decrease with higher levels of SNPL science quality. Overall, this is supportive of the idea that high-quality science is linked to high-value technology especially when science and technology are produced by different individuals or organizations.

**Fig. 4:**
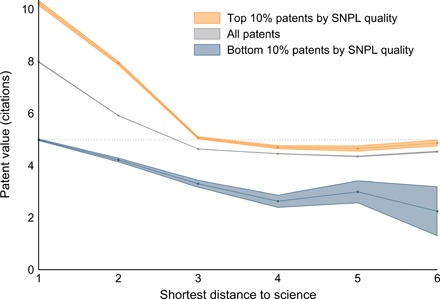
Patent value by distance to the scientific frontier and SNPL science quality. SNPL science quality is the maximum 3-year citation count across scientific publications appearing as SNPL references in a patent. Patent value is measured as the 5-year count of patent forward citations by U.S. patents. Patent value and science quality are residualized using technology field × first filing year FEs. Shaded areas show 95% confidence intervals around the respective means. The distance to the science frontier (*x* axis) is measured as the shortest path to a patent with SNPL references in the patent references network. For patents not at the science frontier, SNPL science quality is the maximum SNPL science quality in patents at the frontier to which they are linked. *N* = 3,709,655.

Our analysis, so far, has focused on patents at the frontier with science, i.e., linked directly to a scientific publication via an SNPL reference. To generalize our findings, we also consider patents connected to scientific publications indirectly via references to other patents. Patents for which the shortest path in the citation network is longer are said to be more distant from the science-technology frontier. Recent studies have used this concept of distance between science and technology and demonstrate that the value of patents monotonically decreases with greater distances from the science frontier (*5*). In Fig. 4, we consider this dimension and describe the value of patents at different levels of distance from the science-technology frontier. We distinguish patents linked (directly or indirectly) to SNPL references at the top 10% and bottom 10% of quality. We also report the average value of all patents at different distances. Patents linked to more than one patent with SNPL references at the same distance are assigned to the patent with the highest-quality SNPL reference.

We find that the correlation between patent value and SNPL science quality largely propagates to patents at higher distances from the science-technology frontier. The increase in patent value for a change from the average patent to patents in the top 10% (patents citing other patents with SNPL references to scientific publications of high quality) is approximately equal to the increase in patent value for moving one step closer to the frontier. For instance, patents that are one step away from the top 10% have about the same value as the average patent at the science frontier. At any distance, patents in the top 10% always have higher average values than those in the bottom 10%. The difference persists also at a substantial distance from the frontier, approximately constant and equal to about a three times higher value. Regression results in the Supplementary Materials confirm that the positive correlation between SNPL science quality and patent value starts fading only after a degree of distance higher than 6. We can conclude that science of high quality spurs technological progress of high value far beyond the science-technology frontier.

In Fig. 3C, we also consider time as a related dimension to distance from science. Time is measured as the lag between the first filing year of a patent family and the publication year of the highest-quality SNPL reference. We study how patent value varies along the SNPL science quality distribution and for different levels of time lag. Shorter time lags are always associated with higher patent value. The correlation with SNPL science quality remains strongly positive for all levels of time distance but is stronger for patents with short time distance. As a consequence, at high levels of SNPL science quality, patent value is high on average; however, it also increases sharply for shorter time lags. Conversely, at low levels of SNPL science quality, the marginal effect of time distance is small.

So far, we have measured patent value with U.S. forward patent citations. However, the results are robust across a broad set of alternative measures of patent value. First, we consider the number of citations from the EPO. Second, we adopt two indicators of monetary value, available for a subsample of patents. We use estimates from Kogan *et al.* (*10*), who propose a measure based on abnormal stock market returns at the patent’s grant event as a proxy for its private value. We further obtain inventor survey-based value estimates of patented inventions from the PatVal survey (*11*). These two measures are only available for a limited sample of patents of about 899,000 and 11,000, respectively. Third, we measure patent scope by the length of the text of the first independent claim. This relies on evidence showing that longer descriptions of the claimed invention imply narrower legal protection and, therefore, a lower patent value (*12*). We consider separately, and when available, the length of the first independent claim in the patent grant publication at the USPTO or the EPO. Table 1 reports descriptive statistics based on the average of all these alternative patent value indicators for patents without SNPL references and for patents in the top 10% and bottom 10% of the SNPL science quality. We replicate regression results for all these alternative measures of patent value in the Supplementary Materials.

**Table 1:** SNPL science quality and alternative measures of patent value. The table presents descriptive statistics for all considered measures of patent value. It reports average values for patents without SNPL references, with SNPL references in the bottom 10%, and with patents in the top 10% of science quality. Patent value and science quality are residualized using technology field × year FEs. Elasticities from corresponding regression analyses are available in the Supplementary Materials.

	**No SNPL**	**Bottom 10%**	**Top 10%**
U.S. citations			
**Mean**	**5.125**	**4.928**	**10.175**
SE	(0.004)	(0.022)	(0.058)
*N*	3,471,621	84,406	84,808
EP citations			
**Mean**	**0.947**	**0.750**	**2.078**
SE	(0.001)	(0.012)	(0.016)
*N*	3,471,621	84,406	84,808
Kogan *et al.* (*10*) (USD)			
**Mean**	**13.326**	**12.517**	**16.704**
SE	(0.044)	(0.625)	(0.469)
*N*	700,613	8866	13,811
PatVal (EUR)			
**Mean**	**11.929**	**8.277**	**24.450**
SE	(0.451)	(3.226)	(4.992)
*N*	8507	349	227
U.S. claim length			
**Mean**	**185.532**	**179.467**	**178.012**
SE	(0.082)	(0.456)	(0.496)
*N*	1,956,651	65,921	69,939
EP claim length			
**Mean**	**143.905**	**140.782**	**129.188**
SE	(0.084)	(0.335)	(0.456)
*N*	1,159,049	42,534	29,972

The relationships discussed are backed up by econometric models that allow for quantifying their average magnitude, assessing their statistical significance, and controlling for the relevant confounding factors. In the Supplementary Materials, we control for narrower technology fields, variation over time across scientific fields, patent applicants, and patent characteristics. Narrower technology field controls and science field controls leave the results essentially unchanged. Applicant characteristics explain about one-third of the baseline correlation, whereas the additional patent-level controls explain about half of the correlation. Qualitatively, the results remain unchanged. Note that the underlying econometric models help rule out alternative explanations but may also lead to overly conservative estimates. In particular, some of the patent-level controls might constitute pathways in which science quality contributes to patent value; thus, including them underestimates the effect.

Furthermore, the Supplementary Materials present estimates across different technology areas and scientific fields. We estimate separate regressions on subsamples defined by the patent’s main technology area and the SNPL scientific area, respectively. The estimates remain strongly significant and comparable across all groups. The effect sizes are larger for patents in chemistry and mechanical engineering compared to electrical engineering and instruments, and for SNPL references in chemistry and physics compared to biology, computer science, medical science, and electrical engineering.

## CONCLUSION

The quality of scientific contributions is often measured in terms of their impact within the scientific community. However, scientific work increasingly needs to be gauged by and acknowledged for its contributions to society and future technical and social advancements. The fact that science quality is practically defined within the realm of science itself contributes to a perception of science as being an independent upstream activity, at times detached from technological progress, with an indirect and delayed impact on society at best.

On the contrary, our study suggests that such an interpretation of the relationship between science quality and technology would largely be a misconception. We show that excellent science is directly linked to inventions of particularly high value. More specifically, our findings demonstrate that there is a robust and strong relationship between the scientific quality of a publication referenced in a patent and the patent’s impact and commercial value.

High-quality science and high-value technology concentrate on the science-technology frontier (*5*). However, it remains unclear whether they directly relate to each other. On the one hand, high-quality science may be hard to translate and may yield mostly low-quality patents. On the other hand, applied science with little scientific impact may lead to outstanding technological results. We rule out this possibility by showing that the positive relationship between science and technology quality is a key mechanism at play at the frontier.

Our results are descriptive, and the exact causes of the strong correlation will have to be analyzed in future work. At this point, it seems most reasonable to presume that industrial users of scientific insights scan the science sector for novel results and use the ones that are most promising for applications in their industrial fields. We doubt that they do so merely on the basis of science citation counts or impact measures. Rather, we expect that they apply their own complex logic and assessments and that they may even avoid using the classical metrics of the science sector altogether. Commercial investments are unlikely to be made on the premise that the citation-measured interest in the scientific community is sufficiently high. Hence, the high correlation between quality measures used in the science sector and those used in the commercial (patent) realm is fortuitous. They are highly unlikely to reflect a spurious selection result.

Putting aside the exact causal links, our results provide intriguing evidence for the governance system of science, e.g., at universities and public research organizations, as well as for funding agencies and science policymakers. The current system steers researchers to strive for success measured in terms of citations and impact. According to our findings, the outcomes of such a system are well aligned with later stages of technology development and translation of science results. Our study does not provide evidence on the optimality of the alignment. However, it contradicts the notion that the application of scientific criteria in science funding decisions leads researchers to engage in exercises that are of little value to society at large. Quite to the contrary, science quality (as measured by scientists) is a strong predictor of applicability and practical value of the technologies developed as the fruits of scientific endeavors. Somewhat paradoxically, when making commercial investment decisions, considering academic measures such as citation counts or impact factors may not be a bad idea.

## MATERIALS AND METHODS

### Scientific literature data

The scientific literature data come from 43 million scientific publications corresponding to all research articles indexed in the Clarivate Analytics WoS database that were published between 1980 and 2016. WoS is the largest bibliographic database of scientific literature and provides all main information for each scientific publication, including authors, affiliations, research fields, and citations (see the Supplementary Materials for further details).

### Patent data

The main source of patent data in our study is DOCDB, a database maintained and updated on a weekly basis by the EPO. It includes records from more than 90 patent offices. We based our study on a sample of more than 4.8 million patent families in DOCDB, comprising all patent families with at least one grant publication at the EPO or the USPTO, with first filing date between 1985 and 2012 included. We included references generated during the search and examination phase of patents filed at the EPO, USPTO, or the World Intellectual Property Organization (see the Supplementary Materials for further details).

### SNPL matching methodology

The dataset to link patents to referenced scientific publications is a full match of DOCDB patent data with bibliographic information included in the WoS. The matching consists of three steps: target selection, search, and quality control. During target selection, cleaning steps are undertaken to exclude NPL strings that are not scientific articles or are outside of the available WoS data. For the remaining entries, a search engine is used to look up NPL full-text strings in a full-text index of the complete WoS. The search engine returns a ranked list of match candidates. During the quality control stage, the topmost candidate is examined, and the match quality is judged according to a field-based scoring.

To validate the matching quality, random subsamples of 1000 NPL references for each patent office were drawn. An NPL string is considered a valid target if it can be found in the WoS using a manual search. We evaluated precision and recall, where precision is computed as the share of correct matches out of all matches delivered by the algorithm, and recall is the share of all targets that can be recovered successfully. Of the 27 million references retained as valid targets, 13 million (47.1%) satisfy the chosen quality requirement.

Our units of analysis are DOCDB patent families that typically include multiple references. The final sample contains 948,006 DOCDB patent families from 1985 to 2017 linked to 2,229,581 distinct scientific articles in the time range of 1980–2016 (see the Supplementary Materials for further details).

### Measures of science quality

#### Scientific citations and journal impact factor

For a given publication, we counted the number of citations in a window of 3 years from publication. The journal impact factor is the average annual number of citations to articles published in that journal during the two preceding years (see the Supplementary Materials for further details).

#### Patent-level aggregation of SNPL references

In our sample, for patents with SNPL, there are, on average, 7.2 SNPL references per patent and 64.0% have references to more than one distinct scientific publication. In our main analyses, we defined SNPL science quality as the maximum science quality across publications in SNPL references in a patent. This is based on the notion that the distribution of scientific forward citations is highly skewed. Consequently, the scientific impact of the most highly cited publication, or the journal with the highest journal impact factor, may be more indicative of SNPL overall science quality than the average across publications. For robustness, we also estimated alternative aggregation operators (see the Supplementary Materials for further details).

### Measures of patent value

#### Patent citations

In our main specification, we proxied patent value with the number of forward citations received by the patent. We constructed the count of citations to a patent from the USPTO over a period of 5 years from the first filing date. In robustness analyses, we used the count of citations from the EPO within 5 years from the first filing date. In case of the EPO citation measure, only examiner-supplied citations were considered (see the Supplementary Materials for further details).

#### Patent scope

As an alternative proxy for patent value, we adopted a measure of the patent’s scope. The value of a patent is considered proportional to the scope of its protection concerning a particular technology. The narrower the scope of protection, the lower its value. The text of patent claims tends to be longer for highly specific and narrow patent protection. Our measure is defined as the logarithm of the number of words in the first independent claim in patents (see the Supplementary Materials for further details).

#### Measures of monetary value

We used data provided by Kogan *et al.* (*10*) based on estimated stock market returns to the grant of the patent as a proxy of the private value of the patent grant. Kogan values are only available for patent families with U.S. patent members, where at least one applicant is a publicly listed U.S. company. We further used survey-based assessments of patent value from the research project PatVal (*11*), which is available for a subset of 11,061 patent families (see the Supplementary Materials for further details).

### Regression models

#### Selection of scientific publications into SNPL references

We considered the probability and frequency in which scientific publications appear in SNPL references, as a function of their scientific quality. The regressions take the following form

$$y_{i}=\beta_{\mathrm{cit}}{\mathrm{cit}}_{i}+\sum_{\text{ft}}\beta_{\text{ft}}{\mathrm{SF}}_{\mathrm{fi}}*T_{\mathrm{fi}}+\epsilon_{i}$$ (1)

The dependent variable *y_i_* is a measure of the probability (or frequency) of a scientific publication appearing among the SNPL references. Respectively, the variable is either a binary or a count variable. Count variables were log-transformed with offset 1. We used several variants of these variables.

The main independent variable *cit_i_* is a measure of scientific quality. We measured scientific quality at the publication level as the number of citations received over a 3-year period starting from publication.

The interaction term *SF_fi_* * *T_fi_* are FEs corresponding to the combination of scientific fields and publication years. These FEs control flexibly for mechanical differences in scientific quality and SNPL frequency across different scientific fields and over time within each scientific field (see the Supplementary Materials for further details).

#### Science quality and patent value: Residualized variables

Usage of SNPL references as well as the quality of cited SNPL vary substantially over technological areas as well as over time. We took this into account explicitly with FE control variables. In all figures relating patents to scientific quality, we applied residualization, which brings the graphical display in line with the regression outputs.

We regressed both the SNPL science quality variables and the patent value variables on the full set of technology area × first filing year FEs. The formal model reads *y_i_* = ∑*_ft_*β*_ft_TA_fi_* ∗ *T_ti_* + ϵ*_i_*. This is done in the full sample of patents both with and without SNPL references. Afterward, we calculated the residual variation as ${\hat{\epsilon }}_{i}\equiv y_{i}-{\hat{y}}_{i}=y_{i}-\sum_{\text{ft}}{\hat{\beta }}_{\text{ft}}{T\text{A}}_{\mathrm{fi}}*T_{\mathrm{ti}}$, where $\hat{\epsilon },\hat{y},$ and $\hat{\beta }$ are estimated values. ${\hat{\epsilon }}_{i}=y_{i}-{\bar{y}}_{\text{ft}}$, where ${\bar{y}}_{\text{ft}}$ is the mean within technology area × first filing year group. Therefore, $E[{\hat{\epsilon }}_{i}]=0$, both overall and within each *ft* group. The values plotted in the graphs are ${\hat{\epsilon }}_{i}+\bar{y}$, where $\bar{y}$ is the full-sample mean of *y*.

#### Science quality and patent value: Regression models

We studied the relationship between the presence and the quality of scientific publications referenced in patents and the value of patents. The regressions take the following form

$$\begin{array}{c} y_{i}=\beta_{\text{hasSNPL}}\;{\text{hasSNPL}}_{i}+\beta_{\text{snplQ}}\;{\text{snplQ}}_{i}+\\ +\sum_{\text{ft}}\beta_{\text{ft}}{\mathrm{TA}}_{\mathrm{fi}}*T_{\mathrm{ti}}+\sum_{a}\;\beta_{a}A_{\mathrm{ai}}+\sum_{n}\;\beta_{n}N_{\mathrm{ni}}+\\ +\sum_{r}\beta_{r}R_{\mathrm{ri}}+\sum_{p}\;\beta_{p}P_{\mathrm{pi}}+\sum_{\text{ft}}\;\beta_{\text{ft}}{\mathrm{SF}}_{\mathrm{fi}}*T_{\mathrm{ti}}+\epsilon_{i} \end{array}$$ (2)

The dependent variable *y_i_* is a measure of patent value. In the main specifications and figures, we used the count of citations from the USPTO within the first 5 years after filing. In alternative specifications, we used the count of citations from the EPO, indicators of monetary value, and patent scope as measured by the length of the first independent claim. All dependent variables are in log terms with offset 1. Given the large dataset and the large number of FE groups, nonlinear (count) models could not be considered.

The term has SNPL*_i_* is a dummy equal to 1 if a patent has at least one reference to a scientific publication. The term snplQ*_i_* is the measure of SNPL science quality. We measured scientific quality at the scientific publication level as the number of citations received over a period of 3 years from publication. We defined SNPL science quality as the maximum scientific quality across SNPL references in a patent when more than one is present.

The interaction term *TA_fi_* * *T_ti_* are FEs corresponding to the combination of technological areas and first filing year. These FEs control flexibly for mechanical differences in patent value across different technological areas and over time within each technological area. The term *A_ai_* are FEs for the applicant of the patent. The term *N_ni_* are FEs for the distinct number of inventors listed on the patent. The term *R_ri_* are FEs for the number of patent references. We used individual FEs for each number of references up to the 95th percentile and assigned one dummy for all patents with a higher number of references. The term *P_pi_* are FEs for the number of patent references to scientific publications. We used an individual FE for each number of references up to the number corresponding to the 95th percentile and aggregate in one FE patent with a higher number of references. The term *SF_fi_* * *T_ti_* are FEs corresponding to the combination of scientific fields and first filing year (see the Supplementary Materials for further details).

## Supplementary Material

http://advances.sciencemag.org/cgi/content/full/5/12/eaay7323/DC1

Download PDF

Science quality and the value of inventions

## SUPPLEMENTARY MATERIALS

Supplementary material for this article is available at http://advances.sciencemag.org/cgi/content/full/5/12/eaay7323/DC1 Supplementary Materials and Methods Supplementary Text Fig. S1. Robustness tests of the main specification. Fig. S2. Heterogeneous effects across self-reference status, applicant country, technology area, and science field. Fig. S3. Patent value-science quality relationship over time. Table S1. SNPL and science quality elasticities (intensive and extensive margin, by SNPL definitions/restrictions). Table S2. Patent value and science quality. Table S3. Patent value and science quality (alternative science quality indicators). Table S4. Patent value and science quality (interdisciplinarity). Table S5. Patent value and science quality (by frontier distance). Table S6. Patent value and science quality (by time distance). Table S7. Top-cited science and patents. References (*13*–*41*)
