# Supplementary material for: Science quality and the value of inventions
Source: Sci Adv. 2019 Dec 11;5(12):eaay7323. doi: 10.1126/sciadv.aay7323 (PMC6905866; doi:10.1126/sciadv.aay7323)
Supplement: http://advances.sciencemag.org/cgi/content/full/5/12/eaay7323/DC1 [file supp_5_12_eaay7323__index.html]

Science Advances | Science AdvancesAAASSearchScience AdvancesMenu

## Supplementary Materials

**This PDF file includes:**

- Supplementary Materials and Methods
- Supplementary Text
- Fig. S1. Robustness tests of the main specification.
- Fig. S2. Heterogeneous effects across self-reference status, applicant country, technology area, and science field.
- Fig. S3. Patent value-science quality relationship over time.
- Table S1. SNPL and science quality elasticities (intensive and extensive margin, by SNPL definitions/restrictions).
- Table S2. Patent value and science quality.
- Table S3. Patent value and science quality (alternative science quality indicators).
- Table S4. Patent value and science quality (interdisciplinarity).
- Table S5. Patent value and science quality (by frontier distance).
- Table S6. Patent value and science quality (by time distance).
- Table S7. Top-cited science and patents.
- References (*13*–*41*)

Download PDF

**Files in this Data Supplement:**

- Adobe PDF - aay7323\_SM.pdf
