# Supplementary material for: Science quality and the value of inventions
Source: Sci Adv. 2019 Dec 11;5(12):eaay7323. doi: 10.1126/sciadv.aay7323 (PMC6905866; doi:10.1126/sciadv.aay7323)
Supplement: Download PDF [file aay7323_SM.pdf]

Science Advances
AAAS

advances.sciencemag.org/cgi/content/full/5/12/eaay7323/DC1

Supplementary Materials for

Science quality and the value of inventions

Felix Poege, Dietmar Harhoff*, Fabian Gaessler, Stefano Baruffaldi

*Corresponding author. Email: dietmar.harhoff@ip.mpg.de

Published 11 December 2019, *Sci. Adv.* **5**, eaay7323 (2019)
DOI: 10.1126/sciadv.aay7323

This PDF file includes:

Supplementary Materials and Methods
Supplementary Text
Fig. S1. Robustness tests of the main specification.
Fig. S2. Heterogeneous effects across self-reference status, applicant country, technology area, and science field.
Fig. S3. Patent value-science quality relationship over time.
Table S1. SNPL and science quality elasticities (intensive and extensive margin, by SNPL definitions/restrictions).
Table S2. Patent value and science quality.
Table S3. Patent value and science quality (alternative science quality indicators).
Table S4. Patent value and science quality (interdisciplinarity).
Table S5. Patent value and science quality (by frontier distance).
Table S6. Patent value and science quality (by time distance).
Table S7. Top-cited science and patents.
References (*13–41*)

Supplementary Materials and Methods
Supplementary Text
Data

Here, we briefly introduce the scientific literature and patent data.

Scientific Literature Data

The scientific literature data comes from 43 million scientific publications corresponding to all research articles indexed in the Clarivate Analytics Web of Science (WoS) database that were published between 1980 and 2016. WoS is the largest bibliographic database of scientific literature and provides all main information for each scientific publication, including authors, affiliations, research fields, and citations. More extensive information on the WoS is available at www.webofknowledge.com.

Patent Data

The main source of patent data in our study is DOCDB, a database maintained and updated on a weekly basis by the European Patent Office (EPO), see www.epo.org/searching-for-patents/data/bulk-data-sets/docdb. It includes records from more than 90 patent offices. We base our study on a sample of more than 4.8 million patent families in DOCDB, comprising all patent families with at least one grant publication at the EPO or the United States Patent and Trademark Office (USPTO), with first filing date between 1985 and 2012, included. We include references generated during the search and examination phase of patents filed at the EPO, USPTO, or the World Intellectual Property Organization (WIPO). Note that at the WIPO, there is no grant procedure, and WIPO examinations are typically conducted by the EPO.

DOCDB contains all information digitally available on these patents. An advantage with respect to non-patent literature (NPL) citations data, as compared to other databases, is the availability of enriched xml text comprising separate fields for title, authors, year, journals title, pages, volume, and number. This allows matching this information separately with bibliographic scientific literature information, substantially improving the quality of the match (see section "Linking Scientific and Patent Literature Data").

Whenever we refer to the technology field, we use the classification of International Patent Classification (IPC) codes in the 34 technology fields provided by WIPO, see https://www.wipo.int/edocs/mdocs/classifications/en/ipc_ce_41/ipc_ce_41_5-annex1.pdf. In a robustness test, we use the Cooperative Patent Classification (CPC) of EPO and USPTO, see www.cooperativepatentclassification.org. Using the CPC system, we distinguish >100 levels (3-digit CPC) or >600 levels (4-digit CPC).

In the dataset of scientific publications (1980-2012), we have in total 42,962,463 publications. When excluding social sciences and humanities, the number shrinks to 35,874,824. Of these scientific publications, 2,248,563 are referenced by patents. Excluding social sciences and humanities, the number shrinks to 2,203,035. When excluding self-references, there are 2,079,713 papers referenced as SNPL. When restricting the time horizon for a SNPL reference to five years, there are 1,627,872 unique referenced papers. When excluding social sciences and humanities, 1,597,426 referenced papers remain. When excluding self-references, 1,465,312 papers remain.

In the dataset of patent families (1985-2012), there are in total 6,962,136 patent family - SNPL reference combinations. When considering the main patent offices separately, there are 1,009,466 (EPO) and 6,177,892 (USPTO) combinations, respectively. There are in total 2,229,581 unique SNPL references. When considering the main patent offices separately, there are 575,624 (EPO) and 2,017,631 (USPTO) unique references, respectively. The number of

patent families, with and without SNPL references, totals 4,767,844. At the main patent offices, this number is 1,960,772 (EPO) and 4,442,742 (USPTO). Finally, the number of patent families with SNPL references is 948,006. At the main patent offices, it is 488,270 (EPO) and 917,179 (USPTO). Discrepancies between numbers originate from the different views on the data. The first paragraph also considers SNPL citations from the 1980-1984 range, whereas the second paragraph does not.

Data transformation

Whenever we use logarithmic transformations on variables with natural zero values (e.g., citation counts), we use a $\log(x + 1)$ transformation. When unifying patent attributes at the patent family level, several decisions must be made. For technology fields, we use the modal technology field of member patents. In case of ties, we use the numerically lowest field. When no field classification is available, we drop the patent family. The CPC system, which is used in Table S4, typically comes with information about the main technology class. Additionally, minor technology classes are specified. We disregard the minor technology classes and proceed as described above. When no main/minor distinction is made, we take all specified classes and proceed as described above. When multiple patent value estimates from Kogan et al. (*10*) or PatVal (*11*) are available, we use the highest one. Some variables with extreme values are winsorized. Backward reference counts, the number of times a patent refers to other patents, are winsorized at the 95th percentile. The same is applied for the number of inventors and SNPL references. Lengths of the first independent claim are winsorized at the 1st and 99th percentiles. We assign scientific fields to scientific publications based on the WoS scientific fields codes. In case of multiple fields, we retain the scientific field whose codes are first in the alphabet. We restrict our sample to SNPL citations where the publication year of the scientific article was at or before the first filing year of the patent family.

We identify literature reviews among scientific articles and exclude them from the analysis. Literature reviews are potentially highly cited within the scientific domain, yet citing them in a patent potentially conveys little information about the quality of the patent. We identify literature reviews by the occurrence of the word "review" in the abstract. Indeed, literature reviews are much more frequent among top-cited articles. Among the top 0.1% highly cited scientific articles, up to 10% are literature reviews. We found that excluding reviews leaves our results virtually unchanged, so we opted to exclude them for simplicity.

Methods

Linking Scientific and Patent Literature Data

"Science" usually refers to the creation and organization of knowledge, often in the form of testable hypotheses and predictions regarding natural phenomena. In a stark simplification, academic scientists (who are mostly employed in the public sector) live in a world governed by the quest for making pioneering contributions to knowledge, hence striving for novelty of insight and for a better understanding of fundamental issues (*13, 14*). According to this view, scientists also follow norms of disclosing newly generated knowledge and information in scientific publications. The societal or private benefit from applications is considered less important but also hard to assess directly. In principle, the science could thus be decoupled from the economic pursuit of wealth and monetary gain.

Conversely, "technology" refers to the realm of the artificial and to artifacts that may have, or may have not, been constructed with the help of scientific insights. Technology is defined in the Organisation for Economic Co-operation and Development (OECD) Frascati Manual as the collection of techniques, skills, methods, and processes used when producing goods and services. Applications of new insights are largely brought about by engineers (*15*). Engineers (who mostly work in the private sector) are governed by rules and incentives that are very differ-

ent from those that guide the behavior of scientists. They seek to contribute new technologies, use secrecy to protect the market positions of their employers, and are involved in strategic considerations of market rivalry. Engineers thus turn knowledge into marketable products that then generate monetary returns for owners. This by now classical view of the relationship between science and technology is described, inter alia, by Allen (*15*) and Brooks (*16*).

Initially understood as two distinct and independent realms, science is now viewed to directly facilitate the application of new knowledge (*17*), and science and technology may follow a process of co-evolution (*18*). Science has also been described as a kind of map used in the process of devising new technologies (*19*). This new view acknowledges that the realms of science and commercial technology development overlap and that their relationship is not necessarily a linear one. While universities mostly generate knowledge, they also file patent applications and license intellectual property. Additionally, corporate entities mostly seek to commercialize new products and services, however, they also engage in basic research not immediately tied to product development and in publication of research results.

SNPL references as a measure of knowledge input

We use NPL references to scientific publications (SNPL) as an indicator of the relatedness of a technology, as described in a patent, to scientific contributions, as reported in scientific publications. Numerous studies have proposed patent citations as an indicator of knowledge flows (*20, 21*). While some authors have raised concerns on the validity of this approach for general patent citations (*22, 23*), SNPL references have been consistently found to be more related to actual knowledge flows than other types of references (*24*). In the context of our study, it is not necessary to interpret SNPL references as a direct indicator of knowledge flows: we assume more broadly that a cited scientific paper contains relevant information for the understanding and the development of a technology.

SNPL matching methodology

The dataset we adopt to link patents to cited scientific publications is a full match of DOCDB patent data with bibliographic information included in the WoS. The matching process is documented in detail in the pilot study by Knaus and Palzenberger, which can be accessed at https://pure.mpg.de/rest/items/item_2540157_9/component/file_2553728/content.

Here we present a brief overview.

The matching consists of three steps: target selection, search, and quality control. In the target selection step, cleaning steps are undertaken to exclude NPL strings that are not scientific articles or are outside of the available WoS data. For the remaining entries, a search engine is employed to look up NPL full-text strings in a full-text index of the complete WoS or Scopus content. The search engine returns a ranked list of match candidates. During the quality control stage, the topmost candidate is examined, and the match quality is judged according to a field-based scoring. Only high-quality matches are considered valid matches for the final dataset.

The matching procedure is applied on a first set of roughly 37 million NPL references. 27 million (71.8%) entries were selected as a potential target and linked to WoS entries. However, not all of these constitute a valid match after taking the quality of the match into account. The quality of a match is judged by six quality indicators (year, volume, page(s), first author, journal title, article title). Each of these indicators equals one if the information from the matched scientific article can be found in the NPL citation string. The quality score is the sum of the indicators and ranges from zero to six.

To validate the matching quality, random subsamples of 1,000 NPL references for each patent office were drawn. An NPL string is considered a valid target if it can be found in the WoS using a manual search. We evaluate precision and recall, where precision is computed as the share of correct matches out of all matches delivered by the algorithm, and recall is the share of all targets that can be recovered successfully. Accepting a quality score of three or higher

as high-quality matches, precision scores of 0.99 and recall scores of 0.96 (EPO) and 0.92 (USPTO) can be achieved. With a quality cutoff at four, the precision increases even further, but recall suffers to a greater extent so that the quality cutoff at three is preferred when equal weight is placed on precision and recall. Finally, precision-recall levels of 0.99/0.96 (EPO), 0.99/0.92 (USPTO) and 0.99/0.97 (WIPO) can be achieved. These numbers are based on a manual validation exercise of 1,000 NPL references per office, as reported in the pilot study by Knaus and Palzenberger.

We, therefore, restrict the sample to matches of quality equal to or higher than three. Out of the 27 million references retained as valid targets, 13 million (47.1%) satisfied this quality requirement. Our units of analysis are DOCDB patent families that typically include multiple references.

While the precision and recall scores are high, they only refer to what could have been matched—the content of the WoS. Clearly, not all scientific publications that can be referenced in patents are covered in this database. We assess the extent of this issue and consider the subset of NPL references that cannot be matched to WoS. We attempt a match to an alternative publication database, Scopus, which has larger coverage. This exercise generates 113,340 additional SNPL links to 49,254 Scopus items from 1996-2016. Given that this is less than 2% of the total, for simplicity, we disregard these links in our analysis.

Our final sample contains 948,006 DOCDB patent families with at least one grant publication and at least one matched SNPL reference at any of the patent offices considered here.

This compares well with previous datasets, and in general, constitutes a larger number of observations than previously identified in existing studies. Ahmadpoor and Jones (*5*) use patent data exclusively at the USPTO between 1976 and 2015 where 759,000 patents were found to be directly linked to at least one scientific publication in WoS via an NPL reference. Jefferson et al. (*25*) starts with 11.8 million scientific publications published between 1980 and 2015,

of which roughly 1.2 million are cited in 690,000 patent *families* (1.1 million patents). This database is one of the few cases in which non-US patents are also considered for SNPL linkages. Another early example of this literature would be Narin and Olivastro (*26*), who link one million front-page references of US and EPO patents to scientific articles. Most recently, Marx and Fuegi (https://papers.ssrn.com/abstract=3331686) link US patents from 1926-2018 to scientific papers from 1800-2018, identifying approximately 15.7 million citation links between 1.4 million patents to 2.9 million papers. In comparison, our dataset links 948,006 patent *families* from 1985-2017 to 2,229,581 distinct scientific articles in the time range of 1980-2016. In our dataset, we use front page NPL references (US patents) and citations from WIPO/EPO search reports. Other minor groups of NPL references that enter our dataset are generated in opposition procedures. Importantly, there is another distinct class of NPL references: in-text references. Front page or search report references are references that are specifically collected by applicants and examiners in the respective patent application and prosecution process. In-text references are added within the patent text by inventors or patent attorneys who write the patent applications. Front page references have the advantage of being a curated set, specifically selected for their relevance. On the other hand, in-text references are argued, by some authors, to be more closely related to the scientific content of the patent. In light of the substantially higher complexity of parsing the actual patent texts, we follow most of the existing literature by disregarding in-text citations.

SNPL self-references

We single out SNPL references to scientific publications in which at least one author also figures among the inventors of the patent and one affiliation of the SNPL references overlaps with the list of applicants in the patent. We refer to these categories as SNPL inventor self-references and applicant self-references, respectively. This type of SNPL references reveals links between

patents and scientific publications originating from either the same organization or from the same individuals, or both. The first analyses rely on the full sample of SNPL references. We present results separately for these categories and exclude them in a later stage.

We consider SNPL inventor self-references those that refer to scientific publications where at least one author has the same name of an inventor on the patent. We consider SNPL applicant self-references those that refer to scientific publications in which at least one affiliation overlaps with the list of applicants in the patent. To match applicants with affiliations, we use a list of manually disambiguated organizations (academic institutions and firms) derived from the combination of multiple sources: the Global Research Identifier Database (GRID), the Orbis database, and the EU Scoreboards database. We merge separately applicants in patents and affiliations in scientific publications to these lists using a probabilistic matching algorithm based on training data. We consider an applicant and an affiliation to be the same when they match with the same entity in the list. Note that the two categories of self-references may overlap. Panel C in fig. S1 presents related descriptive statistics.

Related literature on SNPL references

We briefly summarize the literature that has so far discussed the characteristics of SNPL references and their relationship with patent value.

Hicks et al. (*4*) look at all scientific articles published between 1993 and 1995 in journals indexed in the Science Citation Index (SCI) with at least one US author. They find that about 6,600 of these publications were cited in 1997 US-invented patents. The probability of a publication being cited as SNPL depends not only on the publications research field but also on its scientific impact. If a publication belongs to the top 1% most frequently cited publications, it is about nine times more likely to be cited by a US patent than a randomly chosen US publication. In a similar vein, Popp (*27*) finds in green energy technology fields that scientific articles that

are cited frequently by other articles are also more likely to be cited by patents.

Breschi and Catalini (*28*) analyze all patent applications to the EPO registered in the period between 1990 and 2003 within three technology fields (lasers, semiconductors, and biotechnology) and find about 44,000 patents with 18,000 SNPL references altogether. SNPL references are more frequent in biotech and lasers than in semiconductors, presumably due to the larger distance between the semiconductor technology field and science.

Harhoff et al. (*8*) are among the first to analyze the relationship between the value of such patents and the scientific impact of the underlying scientific contributions. They document a positive relationship between patent value and the number of NPL references. The relationship is particularly strong in the technical area of chemicals and pharmaceuticals. Several other authors have explored the role of NPL references as potential determinants of patent value. Branstetter (*7*) uses a random sample of 30,000 US patents from 1983-1986, of which about 4,300 include SNPL. Those patents that cite scientific articles are of significantly higher quality (more claims and forward-citations) than those that do not. Sorenson and Fleming (*29*) link about 17,300 patents from 1990 with about 16,700 non-patent references. Here, patents that cite NPL receive more citations and are cited earlier than other patents. They argue this positive relationship between the forward-citations and science intensity of a given patent is due to knowledge diffusion through the academic publication. Gittelman and Kogut (*9*) explicitly ask, "Does good science lead to valuable knowledge?" in biotechnology. They suggest that "(...) the evolutionary logics that select valuable scientific publications and valuable patents are different, and because of this, influential publications are not more likely to lead to influential patents than other publications." They employ data on the patent and publication portfolios of 116 biotechnology firms and obtain results that largely confirm their hypothesis.

Suzuki (*30*) argues that patented inventions may be assessed with regard to their monetary or technical quality. The presence of references to scientific publications has a strong posi-

tive effect on the technological value of patents but a weak negative effect on their commercial value. The author also points to considerable heterogeneity across technological fields. Fischer and Leidinger (*31*) use data from Ocean Tomo auctions between 2006 and 2009 to approximate auction prices as a function of observable value correlates. They find only weak and imprecisely estimated effects for the number of NPL references. As they point out, patents traded at Ocean Tomo auctions are not representative and are mostly in the IT and IT-related technical fields. Zahringer et al. (*32*) construct a sample of young life science firms and find that higher-quality academic science is associated with patent citations. This relationship is moderated by the respective firm's research activities. Veugelers and Wang (*33*) use all WoS journal articles published in 2001 and all patents from PATSTAT (version 2013b). They find that only about 10% of articles become SNPL. Novel publications are more likely to receive future citations by patents, particularly the 1% highly novel scientific publications. They further find that publications receiving more scientific citations also receive more patent citations.

Sapsalis et al. (*34*) use data on 155 patent families with application dates between 1985 and 1999 at the EPO to model the relationship between citations received by patents and characteristics of the underlying science. They find that NPL self-references (i.e., the inventors are also authors on the referenced scientific publication) to the scientific literature are associated with an increase in forward-citations of a patent. The authors argue that in such cases of highly valuable patents, "the inventors master (and contribute to) the related science-base (as witnessed by their own publications) and decide to codify their tacit knowledge into technological inventions" (*34, p. 1640*).

In the perspective followed by Fleming and Sorenson (*19*), invention is interpreted as the process of search for new and useful configurations of technological components. Science serves as a map, pointing inventors to particularly useful configurations of components. Alternatively, science allows inventors to avoid searching over less productive solutions. However,

these effects are not pertinent across all technologies. Recourse to science may offer little help when inventors work with highly independent components but should generate high returns when the underlying inventive problem is particularly difficult. Using the population of patents granted by the USPTO in May and June of 1990 (16,822 patents), they find that only 2,919 of these patents reference scientific publications. In the empirical analysis, the authors show that references to scientific publications increase forward-citations received by patents with an elasticity of about 10%.

While the results of the studies discussed here are intriguing, they are typically obtained from relatively small samples that are particularly well-suited for the respective studies. Recent studies tend to feature more comprehensive datasets.

The study most closely related to our findings is Ahmadpoor and Jones (*5*), who analyze the network of US patents citing, directly or indirectly SNPL references. They hereby introduce the distance to the science frontier as a metric for science-technology intensity. Science and patent contribution directly at the frontier turn out to be highly valuable. Value declines monotonically with further distance from the frontier.

Mukherjee et al. (*35*) emphasize the importance of the age structure of references. The authors study (separately) scientific publications in the WoS database and patents, but they do not link NPL references to WoS entries. Both for publications and for patents, they detect a “hot spot” defined by the age structure (of backward references) that is correlated with an increase in citations received by the publication or patent.

Measures of Science Quality

Scientific citations

Our main variable of interest is the scientific quality of publications cited in patents. We use measures of science quality based on the count of forward-citations to publications. This is an

established bibliometric indicator of scientific quality. The use of citations is based on the notion that scientists cite publications they consider influential for their own research. Accordingly, it is possible to assume that highly cited publications have a greater impact on follow-on research and represent a meaningful measure of their scientific quality.

For a given publication, we count the number of citations in a window of three years from publication. This raises the issue that some of these citations may happen later than the filing date of the citing patent. In this case, the number of citations received by a publication may be not independent of the patent itself. In our main specifications, we assume for simplicity that the number of citations to the publication remains indeed independent to the patent citation. In robustness analyses, we verify that the core results remain equivalent when excluding patent citations to publications published in the three years before the filing of the patent (results on file with the authors).

Journal impact factor

An alternative measure of science quality is the impact factor of the journal in which the respective publication is published (JIF). In any given year, the impact factor of a journal is the number of citations, received in that year, of articles published in that journal during the two preceding years, divided by the total number of articles published in that journal during the two preceding years. We use JIF indicators available by the inCite Journal Citations Report. A disadvantage of this measure is that, due to the lack of completeness of the necessary information, the data are available only after 1997. Moreover, the JIF constitutes a retrospective measure of quality of the journal that ignores the possible high variance of publication quality within one journal and over time. On the other hand, the JIF has the advantage of being predetermined at the time a publication is published so that it is not subject to concerns about truncation and mechanical correlation with the measure of patent value. We use the JIF as a variable of interest in table S2

(columns 40-63) to show robustness of our results to alternative measures of science quality.

Patent-level aggregation of SNPL references

In our sample, for patents with SNPL, there are on average 7.2 SNPL references per patent, and a considerable share of 64.0% has references to more than one distinct scientific publication. In our main analyses, we define SNPL science quality as the maximum science quality across publications in SNPL references in a patent. This is based on the notion that the distribution of scientific forward-citations is highly skewed. Consequently, the scientific impact of the most highly cited publication, or the journal with the highest JIF, may be more indicative of SNPL overall science quality than the average across publications. For robustness, we also estimate alternative aggregation operators. Table S3 shows the corresponding results.

We apply a coherent criterion to aggregate at the patent level the information regarding the presence of self-references: we consider a patent as having a self-reference if the scientific publication with the highest scientific quality among the SNPL references is a self-reference.

Measures of Patent Value

Patent citations

Our main dependent variable is patent value. In our main specification, we proxy patent value with the number of forward-citations received by the patent. The number of citations is an established, and perhaps the most widely used, measure of patent value, which is highly correlated with other indicators of the technological and economic value of patents (*8, 31, 36*). Patent citations differ substantially from citations in scientific literature. Scientific citations constitute recognition of scientists of the relevance of previous contributions for their own work. In contrast, patent citations, particularly to other patents, perform the legal function of documenting the technological relatedness of a patent to existing prior art with the scope of assessing its novelty and patentability (*24, 37*).

Due to different legal requirements, citations at the EPO and the USPTO differ substantially. EPO patents tend to cite patents that are essential to document the novelty (or lack of novelty) and patentability of the invention; the applicants, in particular, are not required to provide any citation. Indeed, EPO patents are often filed with no initial references, and, when present, the introduction of references by the applicant is arguably more strategic than in other jurisdictions. Applicants at USPTO are expected to report the most extensive list of citations to all possibly relevant patents. Examiner then complement this list. For this reason, we provide analyses where we count EPO and USPTO citations separately. In our main specifications, we use USPTO citations.

We construct the count of citations to a patent from the USPTO over a period of 5 years from the first filing date. The choice of the time window for the count of scientific and patent citations is motivated primarily by pragmatic considerations: we want to ensure a sufficient period so that the number of citations actually reflects the underlying constructs we are interested in, but we want to limit truncation. The difference between the window considered for scientific publications and for patents is also motivated by the fact that patent applications are not instantly published after filing and—as a result—typically receive few citations within the first years, whereas scientific publications are often cited immediately after publication. In robustness analyses, we use the count of citations from the EPO within 5 years from the first filing date. In case of the EPO citation measure, only examiner-supplied citations are considered.

Patent scope

As an alternative proxy for patent value, we adopt a measure of the patent's scope. The value of a patent is considered proportional to the scope of its protection concerning a particular technology. The narrower the scope of protection, the lower its value. The text of patent claims tends to be longer for highly specific and narrow patent protection. In other words, longer descriptions

of a claimed invention are associated with more specific features that are actually objects of the patent protection (*12*). Our measure is defined as the logarithm of the number of words in the first independent claim in patents. Table S2 (columns 48-55) show the corresponding results. Descriptive statistics are available in the main publication.

Measures of monetary value

Patent citations and patent claim length need to be understood as merely indirect measures of a patent's economic value. Moreover, the number of citations is at times considered to also capture the technological and social value of a patent (*38*), which may differ from the private value for the patent owner. Obtaining direct indicators of the monetary private value of patents is a challenging task. Data on this dimension of patent value have limited coverage. To complement the array of patent value indicators in this direction, we adopt two sources of data. First, we use data provided by Kogan et al. (*10*), based on estimated stock market returns to the grant of the patent, as a proxy of the private value of the patent grant. Kogan values are only available for patent families with US patent members where at least one applicant is a publicly listed US company. The data cover exclusively a total of 1,029,987 patent families, of which 229,525 come with SNPL references. Second, we use survey-based assessments of patent value from the research project PatVal (*11*). This is a subsample of 11,061 patent families with at least one EP patent member, of which 2,554 have SNPL references with their first filing year mostly from 2003-2005. Table S2 (columns 56-63) show the corresponding results. Descriptive statistics are available in the main publication.

The Kogan et al. (*10*) patent value measures have been in widespread use since their publication, but in our setting they come with major drawbacks. Much of the private value of the technology will already be incorporated in the stock price, as previous patent publications and grants in other patent systems are informative for investors. The value narrowly captures the

additional value of a patent granted in the US patent system. Any information related to the technological capability of the firm that the patent reveals will not be incorporated in that measure. On the other hand, the measures from Giuri et al (*11*) are based on a survey, but the exact phrasing measures much more precisely the concept of private patent value: "Suppose that on the day in which this patent was applied for, the applicant and you had all the information you have today regarding the value of this and the related patents. In case a potential competitor of the applicant was interested in buying the whole set of patents (the patent family including all national patents derived from it), what would have been the minimum price (in Euros) that the applicant should have demanded?"

Regression analyses

Regression models

Selection of scientific publications into SNPL references

In a first set of analyses, we consider the probability and frequency in which scientific publications appear in SNPL references, as a function of their scientific quality.

The regressions take the following form

$$y_i = \beta_{cit}\, cit_i + \sum_{ft} \beta_{ft}\, SF_{fi} * T_{fi} + \epsilon_i \tag{1}$$

Dependent variable and predictors of interest:

- y_i: The dependent variable is a measure of the probability (or frequency) of a scientific publication appearing among the SNPL references. Respectively, the variable is either a binary or a count variable. Count variables are log-transformed with offset 1. Given the large dataset and the large number of FE groups, nonlinear (count) models are not considered. We employ several variants of these variables.

- cit_i: The main independent variable is a measure of scientific quality. We measure scientific quality at the publication level as the number of citations received over a 3-year period starting from publication (see section "Measures of Science Quality").

FEs:

- $SF_{fi} * T_{fi}$: These are FEs corresponding to the combination of scientific fields and publication years. These FEs control flexibly for mechanical differences in scientific quality and SNPL frequency across different scientific fields and over time within each scientific field. In total, there are 252 scientific field codes supplied by the WoS.

Science quality and patent value: residualized variables

Naturally, usage of SNPL references as well as the quality of cited SNPL varies substantially over technological areas as well as over time. In the regression models below, this is taken into account explicitly with FE control variables. In all figures relating patents to scientific quality, we apply residualization, which brings the graphical display in line with the regression outputs. To do so, we regress both the SNPL science quality variables as well as the patent value variables on the full set of technology area $\times$ first filing year FEs. The formal model reads $y_i = \sum_{ft} \beta_{ft} F_{fi} * T_{ti} + \epsilon_i$. This is done in the full sample of patents both with and without SNPL references. Afterwards, we calculate the residual variation as $\hat{\epsilon}_i \equiv y_i - \hat{y}_i = y_i - \sum_{ft} \hat{\beta}_{ft} F_{fi} * T_{ti}$, where $\hat{\epsilon}$, $\hat{y}$ and $\hat{\beta}$ are estimated values. In fact, $\hat{\epsilon}_i = y_i - \bar{y}_{ft}$, where $\bar{y}_{ft}$ is the mean within technology area $\times$ first filing year group. Therefore, $E[\hat{\epsilon}_i] = 0$, both overall and within each *ft* group. The values plotted in the graphs are $\hat{\epsilon}_i + \bar{y}$, where $\bar{y}$ is the full-sample mean of y. This returns the absolute levels back to what is contextually expected and interpretable.

In plain terms, this strategy removes level effects within technology area $\times$ first filing year groups by subtracting the mean y within groups. The overall level is retained by adding the

overall y mean. The y variable is transformed. Before, it is a deviation from the *within-group* mean. Afterwards, it is a deviation from the *overall* mean.

Science quality and patent value: regression models

In the empirical analysis, we study the relationship between the presence and the quality of scientific publications referenced in patents and the value of patents.

The regressions take the following form

$$\begin{aligned} y_i = \beta_{hasSNPL}\, \mathit{hasSNPL}_i + \beta_{snplQ}\, \mathit{snplQ}_i + \\ + \sum_{ft} \beta_{ft} \mathit{TA}_{fi} * T_{ti} + \sum_{a} \beta_a A_{ai} + \sum_{n} \beta_n N_{ni} + \\ + \sum_{r} \beta_r R_{ri} + \sum_{p} \beta_p P_{pi} + \sum_{ft} \beta_{ft} \mathit{SF}_{fi} * T_{ti} + \epsilon_i \end{aligned} \quad (2)$$

Dependent variable and predictors of interest:

- y_i: The dependent variable is a measure of patent value. In the main specifications and figures, we use the count of citations from the USPTO within the first 5 years after filing. In alternative specifications, we use: the count of citations from the EPO, indicators of monetary value, and patent scope as measured by the length of the first independent claim (see section "Measures of Patent Value"). All dependent variables are in log-terms with offset 1. Given the large dataset and the large number of FE groups, nonlinear (count) models could not be considered.

- $\mathit{hasSNPL}_i$: A dummy equal to 1 if a patent has at least one reference to a scientific publication

- snplQ_i: A measure of SNPL science quality. We measure scientific quality at the scientific publication level as the number of citations received over a period of 3 years from publication. We define SNPL science quality as the maximum scientific quality across

SNPL references in a patent when more than one is present. We test the robustness of the results to alternative aggregation criteria (see Table S3).

FEs:

- $TA_{fi} * T_{ti}$: These are FEs corresponding to the combination of technological areas and first filing year. These FEs control flexibly for mechanical differences in patent value across different technological areas and over time within each technological area.

- A_{ai}: These are FEs for the applicant of the patent.

- N_{ni}: These are FEs for the distinct number of inventors listed on the patent.

- R_{ri}: These are FEs for the number of patent references. We use individual FEs for each number of references up to the 95th percentile and assign one dummy for all patents with a higher number of references. In regressions involving PatVal (EUR) values, the number of available observations is substantially lower. Here, we include only the log-transformed count of backward patent references when estimating the extended specification.

- P_{pi}: These are FEs for the number of patent references to scientific publications. We use an individual FE for each number of references up to the number corresponding to the 95th percentile and aggregate in one FE patents with a higher number of references. Note that $hasSNPL_i$ is collinear and therefore dropped when this set of FEs is used.

- $SF_{fi} * T_{ti}$: These are FEs corresponding to the combination of scientific fields and first filing year. These FEs control flexibly for mechanical differences in patent value across different scientific fields and over time within each scientific fields.

Regression results

Selection of scientific publications in SNPL references

We present first regression results for the probability that a scientific publication appears in SNPL references as a function of its scientific quality. In the first main specification, Table S1, columns 1 and 2, we consider all SNPL references. Second, in columns 3 and 4, we consider exclusively SNPL references within five years from the year of publication. Third, in columns 5 and 6, we consider references within five years and exclusively if they are the SNPL references with the highest scientific quality. In a fourth variant, columns 7 and 8, we consider only SNPL references that are cited for the first time by an applicant, so that each patent applicant-scientific publication pair is counted at most once (*one per applicant*). We provide regression results where academic patents as well as self-references of various types are excluded (columns 9-15). The exclusion of SNPL self-references is practically irrelevant to the results. Overall, we consistently find a positive and significant effect of science quality on the selection of scientific articles into SNPL references.

Main regression results: SNPL science quality and patent value

Table S2 presents regression results for our core findings. It shows elasticity estimates for the main measure of SNPL science quality and each of the alternative measures of patent value as dependent variables. We incrementally include additional sets of controls. All models include the variable $hasSNPL_i$ as a control for the level effect of having at least one SNPL reference. In the first column for each variable, we present results for our baseline specification where we control exclusively for technology field by year pair FEs. In the second column, we add applicant fixed effects. The third column includes all patent level controls as described in the above section "Regression models." The final fourth column again adds applicant FEs.

The baseline elasticity for US citations with field-year fixed effects is 0.082. To test robust-

ness, we include a large set of fixed effects for the number of SNPL references and the number of backward references, as well as the number of inventors. As a consequence, the US citation elasticity drops to 0.037, which is about 50% of the original magnitude. This is an expected result. All the additional fixed effects are plausibly related to the patent value and have the potential to explain a component of the value transmission of science to technology. For example, when there are more references, the chance of encountering a high citation count is larger. Controlling in a fine-grained manner for the number of references removes this effect. Similarly, more inventors could be an indicator of a more valuable project or being closer to the science frontier (*39, 40*). When also including applicant fixed effects, the estimate drops to around 0.027, less than 40% of the original magnitude. Some applicants, for example large corporations or elite universities, may be better at translating scientific results into technological value. Who exactly these players are remains a question for future research. When including applicant fixed effects but not the extended set of fixed effects, the elasticity is 0.058, around 70% of the original magnitude. All in all, the overall correlation that we observe is constituted of several pathways and mechanisms. However, around 30% of the effect size remains unexplained by the broad set of relevant factors we consider.

In Table S2 (columns 25-39), we add more detailed technology areas as well as science field fixed effects. In the baseline specification, we consider 34 technology areas, whereas in the other columns, we distinguish between more than 100 (600), respectively. All fixed effects are interacted with the first filing year of the patent family. Science field fixed effects are derived from the first WoS field code associated with the highest-quality SNPL reference. The estimates stay quantitatively similar, but the most detailed specifications result in even slightly larger effect sizes. As before, the results stay robust when including extended patent-level controls as well as applicant fixed effects. The magnitudes between specifications with different fixed effect levels are highly similar.

Finally, we estimate models in which we use dichotomous transformations of the variables measuring technological value and science quality. We distinguish patents that are the top 20, 10, or 5% most cited patents within their technology field and filing year. We code the independent variables classifying patents as the 20, 10, or 5% patents by SNPL quality within each scientific field and year of publication. The estimates remain comparable and significant for all combinations of these variables (Table S7). The association with SNPL science quality is stronger for higher levels of science quality, from the top 20% to the top 5%.

Alternative measures of SNPL science quality

As a variant to these specifications, we test the robustness of the results to alternative measures of SNPL science quality. In Table S2 (columns 40-63), we use a measure based on the journal impact factor instead of citations. The number of observations is lower because the journal impact factors are only available to us from 1998 onward. Overall, we find very similar results. The only exceptions are the results for the USD values, where the results are unstable in the first two specifications (columns 5 and 11) but remain positive and significant in our last and most complete specification. In Table S3, we use alternative measures of SNPL science quality derived from different criteria of aggregation at the patent level of the scientific quality of multiple scientific publications, when more than one appear in the NPL references of a patent. When c_i is the citation count of SNPL reference i, in our main models we consider the maximum. Alternatively, we also consider the sum ($\sum_i c_i$), average ($\frac{1}{n}\sum_i c_i$), and square root of the sum of squares ($\sqrt{\sum_i c_i^2}$). We find similar results irrespective of the aggregation criterion used. Panel A of fig. S1 graphically shows the results.

Self-references

Panel C of fig. S1 shows the frequency of the occurrence of self-references: between 5 and 10% of all patent families include a self-reference. Most self-references are inventor

self-references (5-10%), whereas applicant self-references are less frequent with 2-4%. The frequency of self-references tends to decrease with the SNPL science quality (although non-monotonically); this tendency is most pronounced at the top.

In the paper, we consider the possibility that self-references drive the results. On the one hand, from a theoretical standpoint, it is interesting to consider whether high-quality science leads to high-value technologies within or outside the boundaries of the organizations in which it is developed. On the other hand, we want to ensure that the results are not driven by highly productive organizations and individuals that perform scientific and technological activities at the same time.

Panel B of fig. S1 replicates the results reported in the paper, separating different categories of self-references. The different groups of self-references behave very similarly. For the sample without self-references, the estimated elasticities are positive and significant in all specifications, and notably larger than in the sample with only self-references (fig. S2, first row). We can conclude that self-references do not drive the overall effects.

Technology areas, scientific areas, year of patent filing, and applicant countries

We analyze the heterogeneity of the estimates, first across applicant origin. We consider the possibility that the effect is driven by intense science usage of particular countries. To do so, we split the sample by the first applicant country and consider China, Europe (EU-28), Japan, South Korea, and the US separately. In fig. S2, second row, we find confirmation that science quality is important in all countries, but particularly so in Europe, the US, and Japan. Overall, the results are consistent across different geographic areas.

Second, we explore differences between technological fields of patents. Figure S2, third row, presents point estimates for separate regressions by main technology area. In line with previous literature (e.g., (*8*)), we find that effects are particularly strong in chemistry. How-

ever, SNPL science quality also matters for electrical engineering, instruments, and mechanical engineering.

Third, we run separate regressions for different scientific areas of SNPL. For this analysis, we aggregate WoS fields codes into broad areas. We assign patents to different scientific areas based on the area of the scientific publication with the highest science quality. Figure S2, fourth and fifth row, reports graphically the corresponding coefficients for the most frequent scientific areas. The differences are not particularly large, and the coefficients remain positive and significant for all groups. The magnitude of the coefficients is larger for chemistry, electrical engineering, and physics compared to other areas.

Finally, we explore the heterogeneity over time based on the first filing year of patents. We decompose the elasticities calculated in Table S2 (column 1/3, 5/7) over time. Figure S3 depicts the corresponding point estimates. We find that after marginally increasing between 1985 and 2000, the extent of the relationship decreases substantially. The decline after 2000 is especially pronounced for the US system. Understanding the reasons for this decline requires further research.

Interdisciplinarity of SNPL references

Here, we explore the role of the interdisciplinarity of science. Previous studies demonstrate the existence of a close connection between novelty and scientific impact and the ability of scientists to successfully recombine knowledge from distinct domains (*33, 35, 41*). In the context of our analysis, we are interested in exploring whether this dimension explains the correlation between SNPL science quality and patent value. We proxy the interdisciplinarity of science with the interdisciplinary journals as captured by the classification of journals in scientific fields in the WoS.

Some field codes refer directly to multidisciplinary research. We tested our results by includ-

ing journals associated with these codes in the sample of interdisciplinary journals or excluding them. This affects the level estimates of patent value for different values of interdisciplinarity but leaves untouched the correlation with SNPL science quality.

We first plot the share of patents with SNPL references to interdisciplinary scientific publications (Panel D of fig. S1) and subsequently the patent value by SNPL science quality of patents with and without SNPL interdisciplinary references (Panel E of fig. S1). The share of patents with interdisciplinary SNPL references is highest for intermediate values of SNPL science quality. Indeed, we find that interdisciplinarity is associated overall with higher patent value, with the exception of patents at the top of the SNPL science quality distribution. The correlation with SNPL science quality remains, in any case, highly positive for both categories. Table S4 presents the underlying regression results.

Distance of SNPL references

Finally, we present regression results for the frontier distance and time distance of SNPL references. The frontier distance is, in a graph of patent and science citations, the minimum number of links required to reach a scientific article (*5*). A patent with SNPL references thus has a distance equal to one and is called a "frontier patent." The properties of the frontier patent are then inherited by the paper off the frontier. If multiple frontier papers exist, the one with the highest SNPL quality is used. The time distance is the difference in years between the first filing year of the patent family and the publication year of the scientific article. We group the time distance by tertiles.

The related results are presented graphically in the paper. Table S5 shows the results relative to the interaction between the frontier distance of the SNPL references, and Table S6 reports the corresponding regression results for the time distance of the SNPL references. In accordance with what is discussed in the paper, we find that patent families at a short distance, by either

dimension, are of particularly high value and tend to show higher elasticities with SNPL science quality. The elasticities remain, in any case, strongly positive and significant also at a relatively high distance.

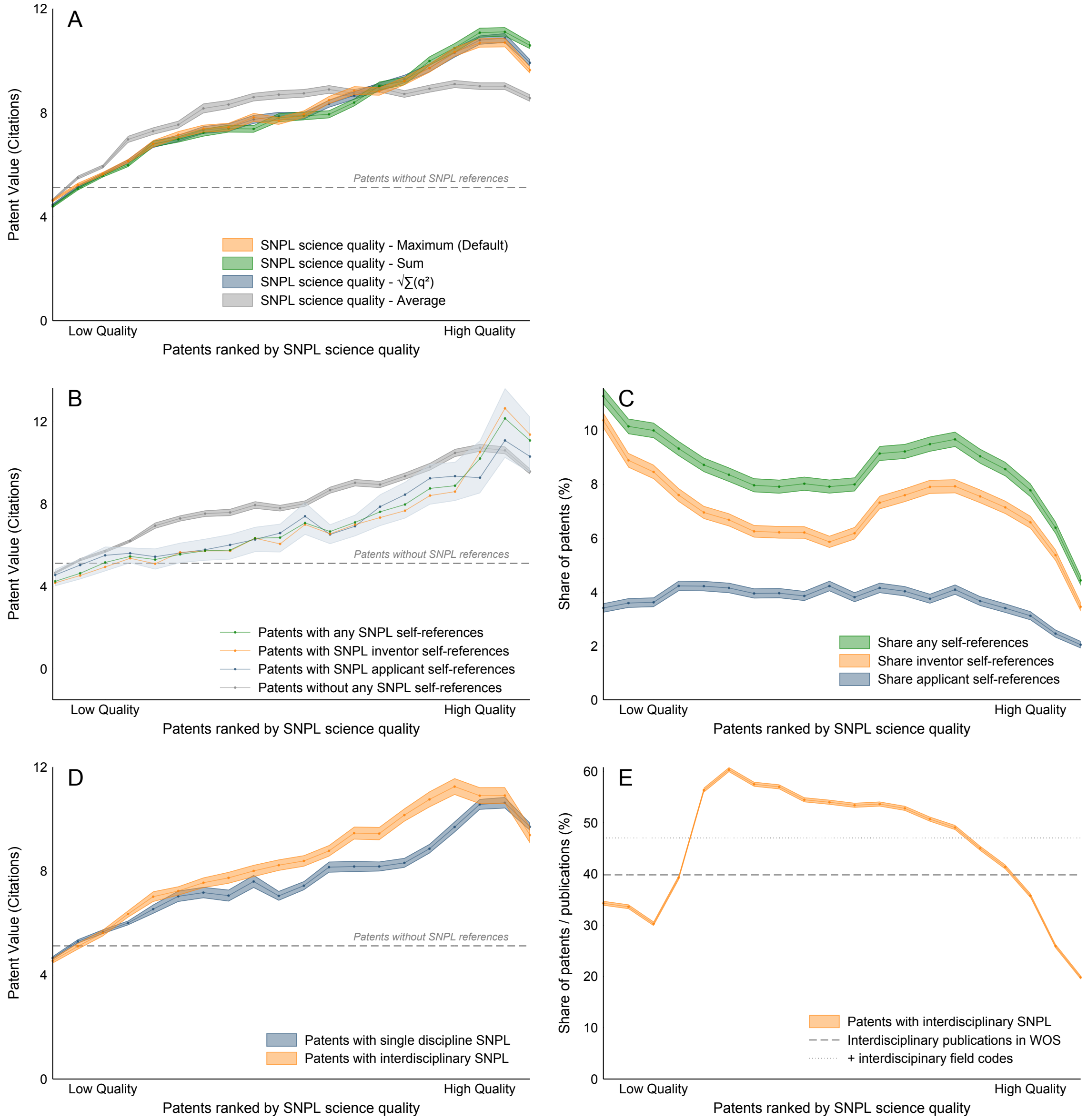

Fig. S1. Robustness tests of the main specification.

Legend figure S1: Separately on the next page.

Legend figure S1: Unless otherwise specified: SNPL science quality is the maximum 3-year citation count across scientific publications appearing as SNPL references in a patent. Patent value is measured as the 5-year count of patent forward-citations by US patents. Patent value and science quality measures are residualized using technology field-first filing year pair FEs. The shaded areas shows 95% confidence intervals around the respective means. The dashed line indicates the average patent value of patents without SNPL references. N = 4,767,844 patents (948,006 with SNPL references).
A: Average patent values by science quality, considering alternative science quality operationalizations. When there are multiple patent-paper references, we by default use the highest-quality reference (orange). In comparison, the average quality also delivers a positive correlation (gray), but it is more diluted. Other aggregation methods that also focus on the top of the distribution are virtually identical to the maximum. These are the sum (green) and the square root of the sum of squares (blue).
B: Average patent value by SNPL science quality and categories of SNPL self-references. The lines show values for any self-reference (green), inventor self-references (orange), and applicant self-references (blue) or patents without SNPL self-references (gray). For visual purposes, the confidence intervals around the means concerning self-references show the maximum extent of the 95% confidence intervals of any of the three underlying measures.
C: Share of SNPL self-references by SNPL science quality.
D: Patent value by SNPL science quality and by interdisciplinarity of SNPL references. Scientific articles are considered interdisciplinary if the journal where they are published is associated with at least two WoS field codes.
E: Share of patents with interdisciplinary SNPL by SNPL science quality.

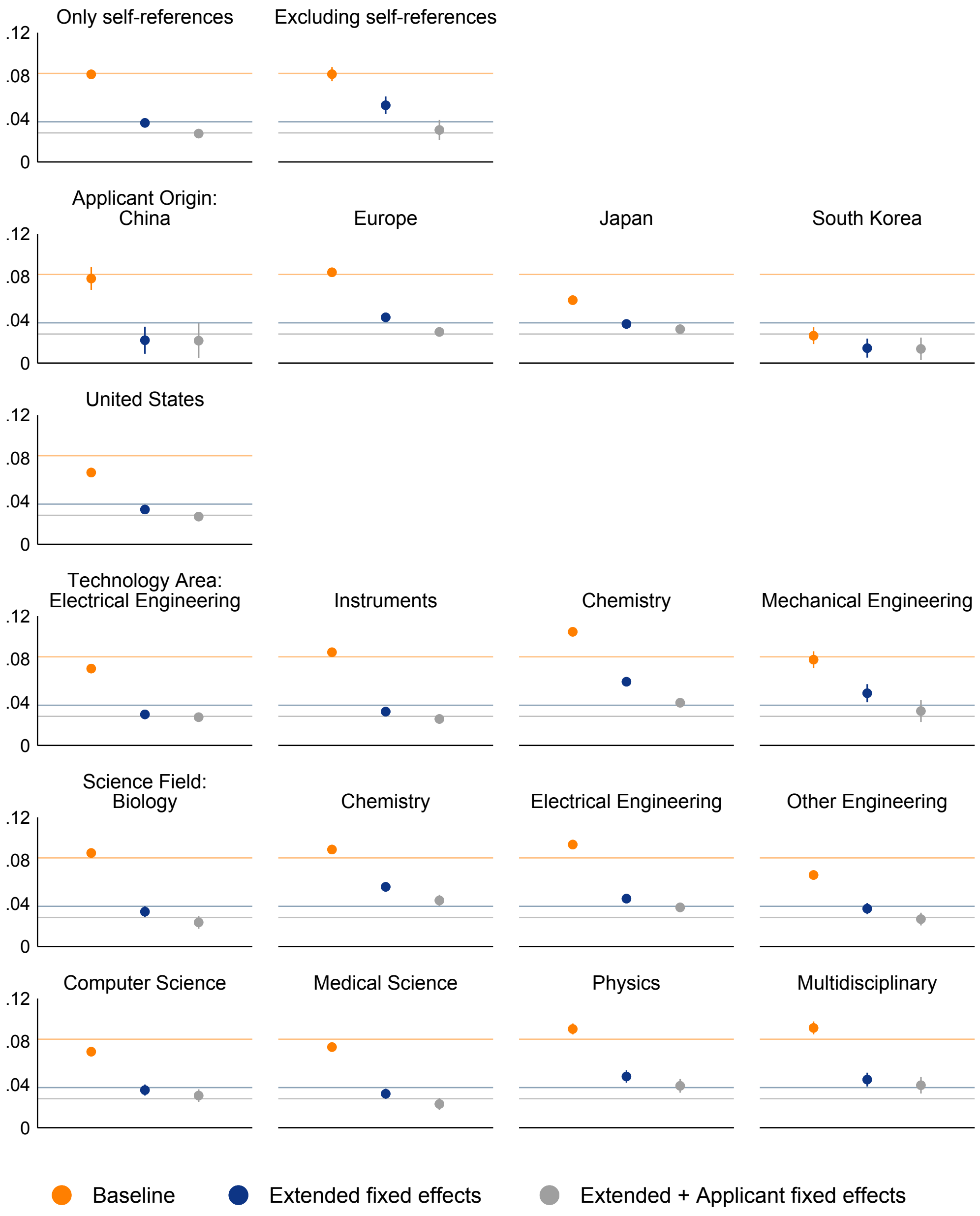

Fig. S2. Heterogeneous effects across self-reference status, applicant country, technology area, and science field.

Legend figure S2: Elasticity estimates and 95% confidence intervals across categories of referenced SNPL are shown. The dependent variables are 5-year US citations (log), and the independent variables are 3-year science citations (log). Estimates are from regressions with SNPL of that category and all non-SNPL patents (cf. Table S2, columns 1, 3, and 4). The lines show elasticity estimates from the full sample of patents without restricting to a particular type of SNPL. The specifications and fixed effects follow the estimation tables below; see, for example, Table S2.

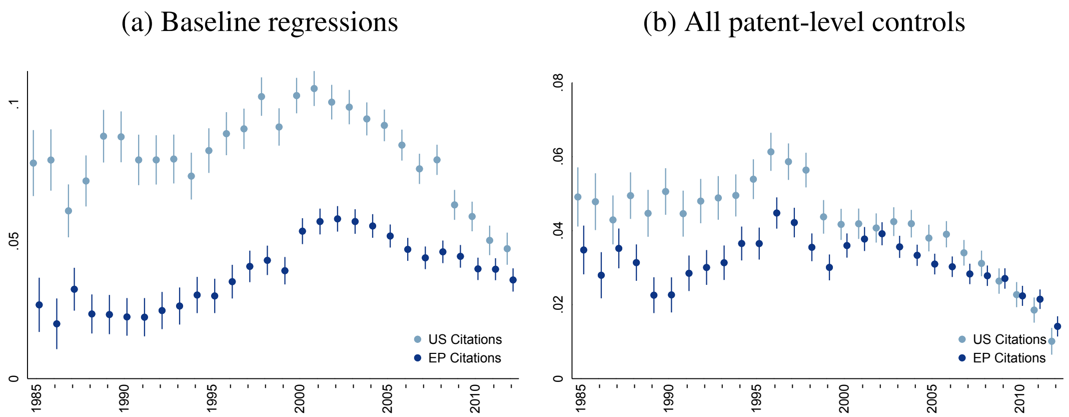

Fig. S3. Patent value-science quality relationship over time.

Legend figure S3: The figure plots the interaction coefficients between each first filing year of patents and SNPL science quality, in a regression with the 5-year patent forward-citations by US patents and EP patents as dependent variables. Science quality is the maximum 3-year citation count of SNPLs of a patent family. Left: Models include technology field and first filing year pair FEs. Right: Models additionally include FEs for SNPL reference counts, patent reference counts, and number of inventors. Range indicators show 95% confidence intervals around the respective regression coefficients.

Table S1. SNPL and science quality elasticities (intensive and extensive margin, by SNPL definitions/restrictions).

SNPL definition	All		Within 5y		Within 5y max quality		One per applicant	
	(1)	(2)	(3)	(4)	(5)	(6)	(7)	(8)
DV: SNPL	(1/0)	Count	(1/0)	Count	(1/0)	Count	(1/0)	Count
3y Cit	0.053	0.068	0.041	0.048	0.012	0.013	0.041	0.043
	(1525.98)	(1551.78)	(1378.07)	(1408.98)	(762.81)	(796.86)	(1371.67)	(1443.36)
Field × Year FE	Yes	Yes	Yes	Yes	Yes	Yes	Yes	Yes
Adj. R-Square	0.113	0.110	0.090	0.089	0.032	0.033	0.089	0.093
Observations	42259668	42259668	42259668	42259668	42259668	42259668	42259668	42259668

	(9)	(10)	(11)	(12)	(13)	(14)	(15)	(16)
SNPL restriction	No academic patents		No applicant self-ref.		No inventor self-ref.		No any self-ref.	
DV: SNPL	(1/0)	Count	(1/0)	Count	(1/0)	Count	(1/0)	Count
3y Cit	0.036	0.046	0.052	0.066	0.050	0.063	0.049	0.063
	(1188.04)	(1195.63)	(1511.65)	(1535.17)	(1476.07)	(1493.49)	(1472.55)	(1489.77)
Field × Year FE	Yes	Yes	Yes	Yes	Yes	Yes	Yes	Yes
Adj. R-Square	0.082	0.077	0.111	0.108	0.107	0.103	0.106	0.103
Observations	42259668	42259668	42259668	42259668	42259668	42259668	42259668	42259668

Legend table S1: Values in “0/1”-columns are semi-elasticities, and values in “Count”-columns are elasticities. Includes WoS subject code times publication year FEs. The level of observation is at the WoS item. Literature reviews are excluded. Science quality (*3y cit*) is measured by 3-year forward-citations by other WoS items. Robust standard errors. T-statistics in parentheses.

Table S2. Patent value and science quality.

DV: Patent 5y Cit (log):	(1)<br>US	(2)<br>US	(3)<br>US	(4)<br>US	(5)<br>EP	(6)<br>EP	(7)<br>EP	(8)<br>EP
3y Cit SNPL ref (max)	0.082	0.058	0.037	0.027	0.042	0.035	0.030	0.023
	(123.56)	(77.19)	(45.97)	(29.75)	(85.49)	(59.33)	(47.64)	(31.70)
Patent-level controls	Base	Base	All	All	Base	Base	All	All
Patent applicant FE	No	Yes	No	Yes	No	Yes	No	Yes
Adj. R-Square	0.156	0.313	0.262	0.362	0.067	0.148	0.100	0.169
Observations	4319660	3764150	4319660	3764150	4319660	3764150	4319660	3764150

DV: Claim Length (log):	(9)<br>US	(10)<br>US	(11)<br>US	(12)<br>US	(13)<br>EP	(14)<br>EP	(15)<br>EP	(16)<br>EP
3y Cit SNPL ref (max)	−0.015	−0.010	−0.012	−0.010	−0.059	−0.036	−0.041	−0.028
	(−26.83)	(−14.28)	(−16.69)	(−11.96)	(−39.08)	(−21.62)	(−20.04)	(−13.02)
Patent-level controls	Base	Base	All	All	Base	Base	All	All
Patent applicant FE	No	Yes	No	Yes	No	Yes	No	Yes
Adj. R-Square	0.157	0.252	0.160	0.253	0.323	0.388	0.325	0.389
Observations	2464729	2099420	2464729	2099419	1241154	1122904	1241154	1122902

DV: Value (log):	(17)<br>(1)<br>USD	(18)<br>(2)<br>USD	(19)<br>(3)<br>USD	(20)<br>(4)<br>USD	(21)<br>(5)<br>EUR	(22)<br>(6)<br>EUR	(23)<br>(7)<br>EUR	(24)<br>(8)<br>EUR
3y Cit SNPL ref (max)	0.021	0.009	−0.047	0.004	0.114	0.101	0.089	0.091
	(6.52)	(6.65)	(−11.47)	(2.23)	(3.69)	(1.79)	(2.88)	(1.61)
Patent-level controls	Base	Base	All	All	Base	Base	All	All
Patent applicant FE	No	Yes	No	Yes	No	Yes	No	Yes
Adj. R-Square	0.113	0.886	0.125	0.887	0.045	0.109	0.065	0.113
Observations	899351	857252	899351	857252	10844	5702	10844	5702

Continued on next page

Table S2. Patent value and science quality. (*continued*)

DV: Patent 5y Cit (log):	(25) US	(26) US	(27) US	(28) US	(29) US
3y Cit SNPL ref (max)	0.082	0.058	0.084	0.086	0.095
	(123.56)	(88.96)	(125.30)	(106.93)	(119.33)
Patent-level controls	Base	SNPL	SNPL	SNPL	SNPL
CPC3 × Year FE		Yes		Yes	
CPC4 × Year FE			Yes		Yes
Science Field × Year FE				Yes	Yes
Patent applicant FE	No	No	No	No	No
Adj. R-Square	0.156	0.152	0.188	0.158	0.191
Observations	4319660	4316956	4316591	4316445	4316080

DV: Patent 5y Cit (log):	(30) US	(31) US	(32) US	(33) US	(34) US
3y Cit SNPL ref (max)	0.037	0.028	0.038	0.047	0.049
	(45.97)	(34.30)	(46.53)	(52.23)	(54.01)
Patent-level controls	All	All	All	All	All
CPC3 × Year FE		Yes		Yes	
CPC4 × Year FE			Yes		Yes
Science Field × Year FE				Yes	Yes
Patent applicant FE	No	No	No	No	No
Adj. R-Square	0.262	0.262	0.286	0.265	0.287
Observations	4319660	4316956	4316591	4316445	4316080

DV: Patent 5y Cit (log):	(35) US	(36) US	(37) US	(38) US	(39) US
3y Cit SNPL ref (max)	0.027	0.023	0.027	0.037	0.037
	(29.75)	(25.05)	(29.46)	(37.09)	(37.45)
Patent-level controls	All	All	All	All	All
CPC3 × Year FE		Yes		Yes	
CPC4 × Year FE			Yes		Yes
Science Field × Year FE				Yes	Yes
Patent applicant FE	Yes	Yes	Yes	Yes	Yes
Adj. R-Square	0.362	0.363	0.373	0.364	0.374
Observations	3764150	3761725	3761213	3761102	3760590

Continued on next page

Table S2. Patent value and science quality. (*continued*)

DV: Patent 5y Cit (log):	(40)<br>US	(41)<br>US	(42)<br>US	(43)<br>US	(44)<br>EP	(45)<br>EP	(46)<br>EP	(47)<br>EP
JIF SNPL ref (max)	0.118	0.079	0.030	0.016	0.087	0.072	0.056	0.044
	(68.01)	(38.54)	(15.80)	(7.15)	(67.05)	(44.37)	(37.49)	(24.40)
Patent-level controls	Base	Base	All	All	Base	Base	All	All
Patent applicant FE	No	Yes	No	Yes	No	Yes	No	Yes
Adj. R-Square	0.148	0.310	0.255	0.359	0.064	0.149	0.097	0.170
Observations	3928677	3385170	3928677	3385170	3928677	3385170	3928677	3385170

DV: Claim Length (log):	(48)<br>US	(49)<br>US	(50)<br>US	(51)<br>US	(52)<br>EP	(53)<br>EP	(54)<br>EP	(55)<br>EP
JIF SNPL ref (max)	–0.034	–0.026	–0.039	–0.031	–0.142	–0.093	–0.111	–0.079
	(–26.18)	(–14.74)	(–25.19)	(–15.45)	(–31.72)	(–17.98)	(–19.98)	(–12.90)
Patent-level controls	Base	Base	All	All	Base	Base	All	All
Patent applicant FE	No	Yes	No	Yes	No	Yes	No	Yes
Adj. R-Square	0.157	0.258	0.160	0.260	0.327	0.393	0.329	0.394
Observations	2289162	1930367	2289162	1930366	1106544	993031	1106544	993031

DV: Value (log):	(56)<br>(1)<br>USD	(57)<br>(2)<br>USD	(58)<br>(3)<br>USD	(59)<br>(4)<br>USD	(60)<br>(5)<br>EUR	(61)<br>(6)<br>EUR	(62)<br>(7)<br>EUR	(63)<br>(8)<br>EUR
JIF SNPL ref (max)	–0.008	0.012	–0.056	0.019	0.212	0.146	0.178	0.143
	(–0.83)	(2.78)	(–5.11)	(3.89)	(2.98)	(1.06)	(2.52)	(1.04)
Patent-level controls	Base	Base	All	All	Base	Base	All	All
Patent applicant FE	No	Yes	No	Yes	No	Yes	No	Yes
Adj. R-Square	0.112	0.890	0.124	0.891	0.045	0.113	0.065	0.117
Observations	773983	734430	773983	734430	10253	5353	10253	5353

Legend table S2: All reported values are elasticities. *3y Cit SNPL ref (max)* is a measure of SNPL science quality corresponding to the maximum 3-year citation count across scientific publications appearing as SNPL references in a patent. Patent-level controls "Base" include technology fields by first filing year pair FEs, and an indicator for the presence of SNPL references. Patent-level controls "All" further include FEs for SNPL reference counts, patent reference counts, and number of inventors. Patent applicant FEs are based on the first applicant on the grant publication. Robust standard errors. T-statistics in parentheses.

Table S3. Patent value and science quality (alternative science quality indicators).

	(1)	(2)	(3)	(4)	(5)	(6)	(7)	(8)
DV (log):	5y Cit US	5y Cit US	5y Cit US	5y Cit US	5y Cit US	5y Cit US	5y Cit US	5y Cit US
SNPL science quality indicators:								
3y Cit SNPL ref (max)	0.037	0.027						
	(45.97)	(29.75)						
3y Cit SNPL ref (sum)			0.038	0.027				
			(46.32)	(29.32)				
3y Cit SNPL ref (avg)					0.042	0.030		
					(47.38)	(29.83)		
3y Cit SNPL ref (sq)							0.038	0.028
							(46.65)	(29.96)
Patent-level controls	All	All	All	All	All	All	All	All
Patent applicant FE	No	Yes	No	Yes	No	Yes	No	Yes
Adj. R-Square	0.262	0.362	0.262	0.362	0.262	0.362	0.262	0.362
Observations	4319660	3764150	4319660	3764150	4319660	3764150	4319660	3764150

Legend table S3: All reported values are elasticities. The table presents results for alternative criteria of aggregation at the patent level of the science quality of SNPL references. The dependent variable is the 5-year count of patent forward-citations by US patents. Patent-level controls “All” include technology fields and first filing year pair FEs, FEs for SNPL reference counts, patent reference counts, and number of inventors. Patent applicant FEs are derived from the first applicant on the grant publication. Robust standard errors. T-statistics in parentheses.

Table S4. Patent value and science quality (interdisciplinarity).

	(1)	(2)	(3)	(4)
DV (log):	5y Cit US	5y Cit US	5y Cit EP	5y Cit EP
Interdisciplinary	0.054	0.046	0.041	0.037
	(15.26)	(12.26)	(15.37)	(12.67)
3y Cit SNPL ref (max) $\times$ Single Discipline	0.043	0.031	0.036	0.028
	(45.33)	(29.77)	(47.83)	(32.40)
3y Cit SNPL ref (max) $\times$ Interdisciplinary	0.034	0.024	0.023	0.018
	(31.47)	(20.68)	(27.86)	(18.73)
Patent-level controls	All	All	All	All
Patent applicant FE	No	Yes	No	Yes
Adj. R-Square	0.262	0.362	0.100	0.169
Observations	4319660	3764150	4319660	3764150

Legend table S4: All reported values are elasticities. *3y Cit SNPL ref (max)* is a measure of SNPL science quality corresponding to the maximum 3-year citation count across scientific publications appearing as SNPL references in a patent. The interdisciplinarity status is taken from the most cited scientific publication appearing as SNPL reference in a patent. Patent-level controls “All” further include FEs for SNPL reference counts, patent reference counts, and number of inventors. Patent applicant FEs are derived from the first applicant on the grant publication. Robust standard errors. T-statistics in parentheses.

Table S5. Patent value and science quality (by frontier distance).

	(1)		(2)	
DV (log):	5y Cit US		5y Cit US	
Distance to frontier:				
1	0.805	(406.42)	0.740	(316.23)
2	0.590	(329.42)	0.562	(288.90)
3	0.358	(195.08)	0.394	(192.48)
4	0.216	(100.75)	0.262	(100.96)
5	0.120	(41.62)	0.190	(50.51)
6	0.093	(25.15)	0.170	(34.30)
7	0.106	(28.47)	0.204	(40.20)
8	0.105	(32.02)	0.199	(45.92)
9	0.095	(30.79)	0.189	(46.96)
10	0.057	(17.84)	0.149	(35.72)
3y Cit SNPL ref (max)	0.060	(206.95)		
3y Cit SNPL ref (max) × 1			0.091	(144.40)
3y Cit SNPL ref (max) × 2			0.073	(155.18)
3y Cit SNPL ref (max) × 3			0.042	(74.00)
3y Cit SNPL ref (max) × 4			0.032	(32.85)
3y Cit SNPL ref (max) × 5			0.013	(8.03)
3y Cit SNPL ref (max) × 6			0.012	(5.41)
3y Cit SNPL ref (max) × 7			0.002	(0.71)
3y Cit SNPL ref (max) × 8			0.001	(0.28)
3y Cit SNPL ref (max) × 9			–0.001	(–0.74)
3y Cit SNPL ref (max) × 10			0.001	(0.39)
Patent-level controls	Base		Base	
Patent applicant FE	No		No	
Adj. R-Square	0.186		0.188	
Observations	4378579		4378579	

Legend table S5: All reported values are elasticities. Patent-level controls "Base" include technology fields by first filing year pair FEs and an indicator for the presence of SNPL references. *3y Cit SNPL ref (max)* is a measure of SNPL science quality corresponding to the maximum 3-year citation count across scientific publications appearing as SNPL references in a patent. Robust standard errors. T-statistics in parentheses.

Table S6. Patent value and science quality (by time distance).

	(1)	(2)	(3)	(4)
DV (log):	5y Cit US	5y Cit US	5y Cit EP	5y Cit EP
SNPL time distance:				
Short	0.139	0.115	0.089	0.076
	(33.16)	(25.42)	(27.95)	(21.57)
Medium	0.052	0.040	0.039	0.028
	(12.11)	(8.50)	(12.10)	(7.89)
3y Cit SNPL ref (max) × Short	0.041	0.032	0.034	0.027
	(37.57)	(26.96)	(39.15)	(28.34)
3y Cit SNPL ref (max) × Medium	0.038	0.027	0.027	0.021
	(33.59)	(21.89)	(30.59)	(20.94)
3y Cit SNPL ref (max) × Long	0.031	0.019	0.028	0.018
	(26.98)	(14.83)	(30.25)	(17.53)
Patent-level controls	All	All	All	All
Patent applicant FE	No	Yes	No	Yes
Adj. R-Square	0.263	0.363	0.101	0.169
Observations	4319660	3764150	4319660	3764150

Legend table S6: All reported values are elasticities. *3y Cit SNPL ref (max)* is a measure of SNPL science quality corresponding to the maximum 3-year citation count across scientific publications appearing as SNPL references in a patent. *Short*, *Medium*, and *Long* time distance are dummies for the tertiles of time distance. Patent-level controls "All" further include FEs for SNPL reference counts, patent reference counts, and number of inventors. Patent applicant FEs are derived from the first applicant on the grant publication. Robust standard errors. T-statistics in parentheses.

Table S7. Top-cited science and patents.

DV: 5y Cit US	(1)<br>log	(2)<br>log	(3)<br>log	(4)<br>Top 20%	(5)<br>Top 20%	(6)<br>Top 20%
Top 20% Science	0.053	0.028	0.016	0.010	0.004	0.004
	(14.54)	(8.19)	(4.43)	(5.81)	(2.25)	(2.42)
Top 10% Science	0.050	0.028	0.023	0.025	0.014	0.009
	(11.02)	(6.82)	(5.21)	(12.02)	(7.08)	(4.19)
Top 5% Science	0.176	0.074	0.057	0.069	0.029	0.023
	(48.33)	(21.63)	(15.35)	(40.34)	(17.58)	(12.27)
Patent-level controls	Base	All	All	Base	All	All
Patent applicant FE			Yes			Yes
Adj. R-Square	0.155	0.262	0.362	0.020	0.095	0.172
Observations	4319660	4319660	3764150	4321415	4321415	3765838

DV: 5y Cit US	(7)<br>Top 10%	(8)<br>Top 10%	(9)<br>Top 10%	(10)<br>Top 5%	(11)<br>Top 5%	(12)<br>Top 5%
Top 20% Science	0.005	0.000	0.002	0.000	–0.002	0.001
	(3.60)	(0.22)	(1.60)	(0.33)	(–2.14)	(1.03)
Top 10% Science	0.019	0.009	0.004	0.016	0.008	0.002
	(10.87)	(5.44)	(2.30)	(11.60)	(6.13)	(1.66)
Top 5% Science	0.051	0.019	0.016	0.032	0.010	0.009
	(35.95)	(14.01)	(10.53)	(28.86)	(9.37)	(7.66)
Patent-level controls	Base	All	All	Base	All	All
Patent applicant FE			Yes			Yes
Adj. R-Square	0.021	0.088	0.161	0.023	0.082	0.152
Observations	4321415	4321415	3765838	4321415	4321415	3765838

Legend table S7: Shows indicators of top-cited science and top-value patents as independent and dependent variables, respectively. The variable "Top 20% Science" is equal to one if the referenced SNPL is in the top 20% of the scientific literature of the same field and publication year. When the SNPL is in the top 5%, it is also in the top 10% and top 20%; thus, the estimates have to be added up to get the full effect. The reference groups are SNPL in the bottom 80%. Columns 1-3 use log 5-year US citations as dependent variable; coefficient estimates are semi-elasticities. The other columns use indicators of the patent being in the top 20% (10, 5) of the technology area in the same year. In columns 4-12, linear probability models are used, and coefficient estimates are increases in probability. The specifications are the same as in Table S2.
